# The Value of Stereotactic Radiotherapy After FOLFIRINOX in Patients with Pancreatic Cancer with Vascular Contact—A Nationwide, Retrospective Cohort Study

**DOI:** 10.3390/cancers18040700

**Published:** 2026-02-20

**Authors:** Freek R. van ‘t Land, Leonard W. F. Seelen, Maaike Verheij, Thomas F. Stoop, Olivier R. Busch, Marc G. H. Besselink, Lois A. Daamen, Marcel den Dulk, Sebastiaan Festen, Ignace H. J. T. de Hingh, Marjolein Y. V. Homs, Martijn P. W. Intven, Daan J. Lips, Maartje Los, Vincent E. de Meijer, Joost J. Nuyttens, Martijn W. J. Stommel, Roeland F. de Wilde, Johanna W. Wilmink, I. Quintus Molenaar, Hjalmar C. van Santvoort, Bas Groot Koerkamp, Casper H. J. van Eijck

**Affiliations:** 1 Department of Surgery, Erasmus MC Cancer Center, 3015 Rotterdam, The Netherlands; 2 Department of Surgery, Regional Academic Cancer Center Utrecht, UMC Utrecht Cancer Center, St. Antonius Hospital Nieuwegein, Utrecht University, 3584 Utrecht, The Netherlands; 3 Department of Surgery, Amsterdam University Medical Center, University of Amsterdam, 1105 Amsterdam, The Netherlands; 4 Cancer Center Amsterdam, 1105 Amsterdam, The Netherlands; 5 Division of Surgical Oncology, Department of Surgery, University of Colorado Anschutz Medical Campus, Aurora, CO 80045, USA; 6 Department of Surgery, Maastricht University Medical Center, 6229 Maastricht, The Netherlands; 7 Department of General, Visceral and Transplant Surgery, University Hospital Aachen, 52074 Aachen, Germany; 8 NUTRIM-School of Nutrition and Translational Research in Metabolism, Maastricht University, 6229 Maastricht, The Netherlands; 9 Department of Surgery, OLVG, 1105 Amsterdam, The Netherlands; 10 Department of Surgery, Catherina Hospital, 5623 Eindhoven, The Netherlands; 11 Department of Medical Oncology, Erasmus MC Cancer Center, 3015 Rotterdam, The Netherlands; 12 Department of Radiotherapy, University Medical Center Utrecht, 3584 Utrecht, The Netherlands; 13 Department of Surgery, Medisch Spectrum Twente, 7512 Enschede, The Netherlands; 14 Department of Medical Oncology, Regional Academic Cancer Center Utrecht, UMC Utrecht Cancer Center, St. Antonius Hospital Nieuwegein, Utrecht University, 3584 Utrecht, The Netherlands; 15 Department of Surgery, University of Groningen and University Medical Center Groningen, 9713 Groningen, The Netherlands; 16 Department of Radiation Oncology, Erasmus MC Cancer Center, 3015 Rotterdam, The Netherlands; 17 Department of Surgery, Radboud University Medical Center, 6525 Nijmegen, The Netherlands; 18 Department of Medical Oncology, Amsterdam University Medical Center, University of Amsterdam, 1105 Amsterdam, The Netherlands

**Keywords:** locally advanced pancreatic cancer, pancreatic cancer, stereotactic radiotherapy, FOLFIRINOX chemotherapy, overall survival

## Abstract

Background/Objectives: Stereotactic body radiotherapy (SBRT) aims to prolong overall survival (OS) in patients with pancreatic ductal adenocarcinoma (PDAC) with vascular contact without progression of disease after (m)FOLFIRINOX. The primary objective of this study was to determine the potential value of SBRT. Methods: This nationwide, retrospective cohort study included patients with PDAC without progression of disease after at least four cycles of (m)FOLFIRINOX. The study comprised two cohorts, the SBRT and the No SBRT group. A landmark analysis excluded patients with a follow-up or OS time less than 12 months to minimize immortal time bias in the SBRT group. The primary outcome was OS from diagnosis. Secondary outcomes were the histopathological characteristics after resection. Results: Overall, 331 patients were included, of whom 231 were in the landmark analysis. In the overall cohort, the median OS was 20.7 months in the SBRT group versus 15.7 months in the No SBRT group (*p* = 0.004). In the landmark analysis, the median OS was 23.2 months in the SBRT group compared to 22.3 months in the No SBRT group (*p* = 0.554). These results indicate the presence of immortal time bias in the overall cohort in favor of the SBRT group. In the subgroup after resection, ypT0-2 (95% versus 76.5% [*p* = 0.026]), ypN0 (75% versus 37.3% [*p* < 0.004]), and absence of perineural invasion (50% versus 68.6% [*p* = 0.015]) were more prevalent in the SBRT group. Conclusions: In a landmark analysis, including only patients who survived at least 12 months after diagnosis, we found no difference in median OS between (m)FOLFIRINOX-only and (m)FOLFIRINOX with consecutive SBRT.

## Simple Summary

Pancreatic cancer with vascular contact without distant metastasis is treated with chemotherapy. In addition, stereotactic radiotherapy has been used following chemotherapy in an attempt to increase progression-free and overall survival. However, the benefit of radiotherapy in improving local disease control or overall survival is not known. In the current study, in patients who did not develop progressive disease after at least four cycles of (modified)FOLFIRINOX, stereotactic radiotherapy was not associated with improved overall survival compared to chemotherapy-only treatment. In the subgroup of patients who underwent resection, stereotactic radiotherapy was associated with favorable histopathological characteristics.

## 1. Introduction

Pancreatic ductal adenocarcinoma (PDAC) is a lethal malignancy with a five-year overall survival (OS) rate of approximately 11% for all stages of the disease [1,2]. About 40% of patients with PDAC are diagnosed with tumors that have vascular contact without detectable distant metastases on cross-sectional imaging [3]. In these cases, upfront surgical resection is associated with high morbidity. Most importantly, these patients have a high risk of early postoperative recurrence, and OS is poor [4]. Therefore, the National Comprehensive Cancer Network (NCCN) guidelines recommend neoadjuvant/induction chemotherapy with multi-agent chemotherapeutic regimens [5].

If patients with PDAC with vascular contact show adequate disease control following systemic treatment, eligibility for surgical exploration and possible tumor resection should be explored. Stereotactic body radiotherapy (SBRT) has been performed preoperatively, considering that data from multiple non-randomized studies suggest an improvement in local tumor control and an increase in margin negative (R0) resections following SBRT treatment [6,7,8,9,10,11,12,13]. However, since these were non-randomized, single-armed studies, the risk of inherent selection bias was high. More recently, a randomized trial in patients with borderline resectable pancreatic cancer (BRPC) investigated the potential added value of SBRT after treatment with neoadjuvant (m)FOLFIRINOX. It found worse OS after SBRT treatment [14]. Statistical inference on efficacy was, however, impossible because the proportion of R0 resections in the SBRT group did not meet the predetermined threshold; therefore, patient accrual in the SBRT arm was terminated prematurely. This previous study challenges the use of SBRT in pancreatic cancer. However, for patients ineligible for surgical resection, SBRT is considered a suitable option to potentially improve local tumor control. Treatment with SBRT is generally considered safe, although serious gastrointestinal bleedings have been reported [11,12,15].

The present study aimed to determine the potential added value of SBRT treatment in patients with PDAC with vascular contact without disease progression after at least four cycles of (m)FOLFIRINOX.

## 2. Materials and Methods

### 2.1. Study Design

This nationwide, retrospective, observational cohort study was conducted according to the Strengthening of Observational Studies in Epidemiology guidelines [16]. Data from two cohorts were analyzed. The first cohort comprised patients from the prospective, maintained Dutch LAPC registry, in which patients with locally advanced pancreatic cancer (LAPC) were included between 2015 and 2019 at 15 Dutch hospitals affiliated with the Dutch Pancreatic Cancer Group [17]. Patients in this cohort who had been treated with (m)FOLFIRINOX without consecutive SBRT were assigned to the No SBRT group. The second cohort comprised patients who were treated with (m)FOLFIRINOX followed by consecutive SBRT and were assigned to the SBRT group. These patients received SBRT between 2015 and 2020 at the Erasmus MC Cancer Institute. Most patients were referred to Erasmus MC for SBRT after completion of systemic chemotherapy and restaging. A proportion of the patients had participated in one of two clinical studies on SBRT [11,12]. The medical ethics review board of Erasmus MC Rotterdam (MEC-2022-0279) approved the analyses presented in this study (approval date: 13th April 2022). The study was approved as non-WMO research. Therefore, no informed consent was obtained.

### 2.2. Patients

For this study, inclusion criteria were (1) pathology-proven PDAC, (2) BRPC or LAPC according to the NCCN guidelines [5], and (3) treatment with (m)FOLFIRINOX. Patients who underwent a staging laparoscopy were only included in this study if no metastases were found. The staging laparoscopy was performed at the time of diagnosis, not during restaging after (m)FOLFIRINOX treatment. Exclusion criteria were (1) resectable or metastatic PDAC, (2) treatment with <4 cycles of (m)FOLFIRINOX, (3) RECIST progressive disease at restaging between 4 and 8 cycles [18], (4) no data on radiotherapy treatment or treatment with a type of radiotherapy other than SBRT, and (5 other localized ablative therapies (e.g., radiofrequency ablation or irreversible electroporation). Regarding exclusion criterion 3, patients were excluded if disease progression had occurred at restaging after (m)FOLFIRINOX because progression would have precluded them from local treatment such as SBRT.

### 2.3. Outcomes

The primary outcome was the median OS (mOS). Secondary outcomes were the histopathological characteristics of the patients who underwent a resection.

### 2.4. Treatments

#### FOLFIRINOX or Modified FOLFIRINOX

The FOLFIRINOX regimen consists of a bolus of 400 mg/m^2^ 5-fluorouracil, followed by 85 mg/m^2^ oxaliplatin, 400 mg/m^2^ leucovorin, and 180 mg/m^2^ irinotecan. This is immediately followed by 2400 mg/m^2^ 5-fluorouracil, given by a continuous intravenous infusion over a period of 46 h. This regimen is repeated every 14 days, and one bi-weekly administration is defined as one cycle. In the case of modified FOLFIRINOX, the bolus of 5-fluorouracil is not given, and the dose of irinotecan is reduced to 150 mg/m^2^.

Stereotactic Body RadiotherapySBRT is delivered with the CyberKnife^®^ (Accuray, California, USA). A total dose of 40 Gray is administered over a period of five consecutive days (5 × 8 Gray). Patients were positioned in a supine posture for radiation treatment. Prior to the procedure, they were prepared using a specialized CT scanner in the treatment position with an immobilization device. To monitor the movement of the bowel and stomach during each treatment session, a CT scan was performed at the end of expiration just before each treatment fraction.

The clinical target volume (CTV) was defined as the gross tumor volume (GTV) along with a 5 mm margin to account for potential tumor spread. The planning target volume (PTV) included the CTV with an additional 2 mm margin. Dose constraints for surrounding organs at risk were set as follows: a maximum of 50 Gy in equivalent dose (EQD2) at 2 Gy per fraction (a/b = 3) to the spinal cord, and a limit of 35 Gy in 5 fractions to the stomach and bowel (a/b = 3). The mean dose to the kidneys was not allowed to exceed 18 Gy in EQD2 (a/b = 2.5), and no more than 700 cc of the liver could receive an absolute dose greater than 20 Gy.

### 2.5. Data Collection

Predefined patient characteristics, disease characteristics, treatment details, and clinical and pathological outcomes were collected for all patients independent of the cohort. Data from the SBRT group were requested from the Netherlands Comprehensive Cancer Organisation (IKNL). If possible, missing data in the IKNL database were retrieved from local electronic patient files. Data from the No SBRT group were retrieved from the Dutch LAPC registry. Case report forms were equal for every patient independently.

### 2.6. Definitions

The performance status was scored according to the Eastern Cooperative Oncology Group (ECOG) definition [19]. Based on the radiology reports of expert pancreaticobiliary radiologists, tumors were classified as BRPC or LAPC according to the National Comprehensive Cancer Network guidelines version 2.2023—19 June 2023 [5]. Tumor–node–metastasis (TNM) staging was scored according to the 8th edition of the American Joint Committee on Cancer (AJCC) staging system [20]. Tumor size at the time of diagnosis was measured as the largest diameter on cross-sectional imaging. The margin status was defined as R0 (margin clearance > 1 mm) or R1 (margin clearance ≤ 1 mm) according to the Royal College of Pathologists definition [21].

### 2.7. Statistical Analysis

Baseline variables were analyzed using descriptive statistics. Continuous data are presented as medians with interquartile ranges. Categorical data are shown as counts with percentages. Median follow-up time was calculated using the reverse Kaplan–Meier method. The mOS was calculated from the date of diagnosis until the date of death. In the resected cohort, the mOS was also calculated from the date of resection until the date of death. Patients who were alive were censored at the date of the last follow-up. The median OS time was estimated using the Kaplan–Meier method. Potential variables influencing OS from the time of diagnosis were analyzed using Cox regression analysis. Log minus log plots were used to check the proportional hazards assumption. Variables with a significance level of <0.200 in univariable analysis were investigated in a multivariable analysis. Variables were eliminated using backward selection until the multivariable model only consisted of significant parameters. A landmark analysis was performed, excluding data from all patients with a follow-up or OS time of fewer than 12 months. The 12-month time point was chosen because, on average, SBRT is administered around 6 months after diagnosis. It is, therefore, unlikely that a potential survival benefit from SBRT will be observed earlier than 12 months after diagnosis. An OS difference established earlier is likely subject to immortal time bias, as the progression of disease after chemotherapy excludes a patient from subsequent locoregional treatment. Missing data were not imputed. A *p*-value < 0.050 was considered statistically significant. Analyses were performed with SPSS, version 28, and R, version 4.1.0.

## 3. Results

### 3.1. Patient, Disease and Treatment Characteristics

**Figure 1:**
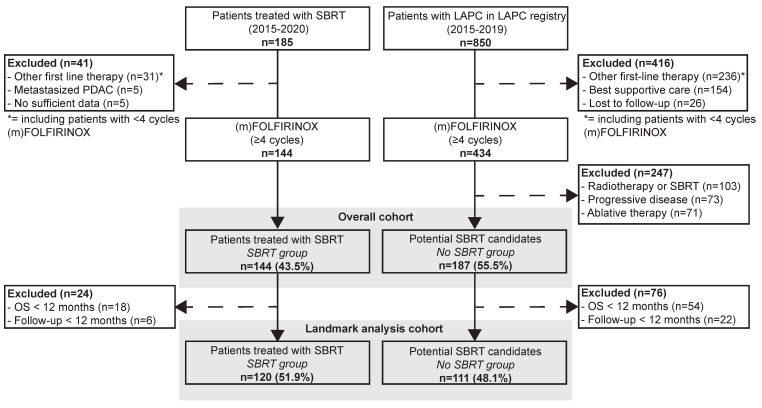
Flow chart of patient selection.

The overall cohort consisted of 331 patients, of whom 231 were included in the landmark analysis cohort, as shown in Figure 1. In the landmark analysis cohort, 120 (51.9%) patients were included in the SBRT group and 111 (48.1%) in the No SBRT group. Of the SBRT group, 27 patients (22.5%) had participated in the LAPC-1 trial, 28 patients (23.3%) had participated in the LAPC-2 trial, and 65 patients (54.2%) had not participated in a clinical trial. In the SBRT group, 36.7% had an ECOG performance score of 0 compared to 52.3% in the No SBRT group (*p* = 0.005). The median baseline CA 19-9 was 356 U/mL in the SBRT group and 268 U/mL in the No SBRT group (*p* = 0.159). In the SBRT group, 62.5% had an LAPC compared to 60.4% in the No SBRT group (*p* = 0.738). In the SBRT group, 37.5% underwent a staging laparoscopy at baseline compared to 2.7% in the No SBRT group (*p* < 0.001). In the SBRT group, 16.7% underwent a resection compared to 45.9% in the No SBRT group (*p* < 0.001). Moreover, the SBRT group received a median of eight cycles of total (m)FOLFIRINOX compared to a median of nine cycles in the No SBRT group (*p* = 0.020). Detailed patient, disease, and treatment characteristics of the landmark analysis cohort and the overall cohort are shown in Table 1 and Table S1

### 3.2. Primary Outcomes

#### 3.2.1. Overall Survival—Overall Cohort

**Figure 2:**
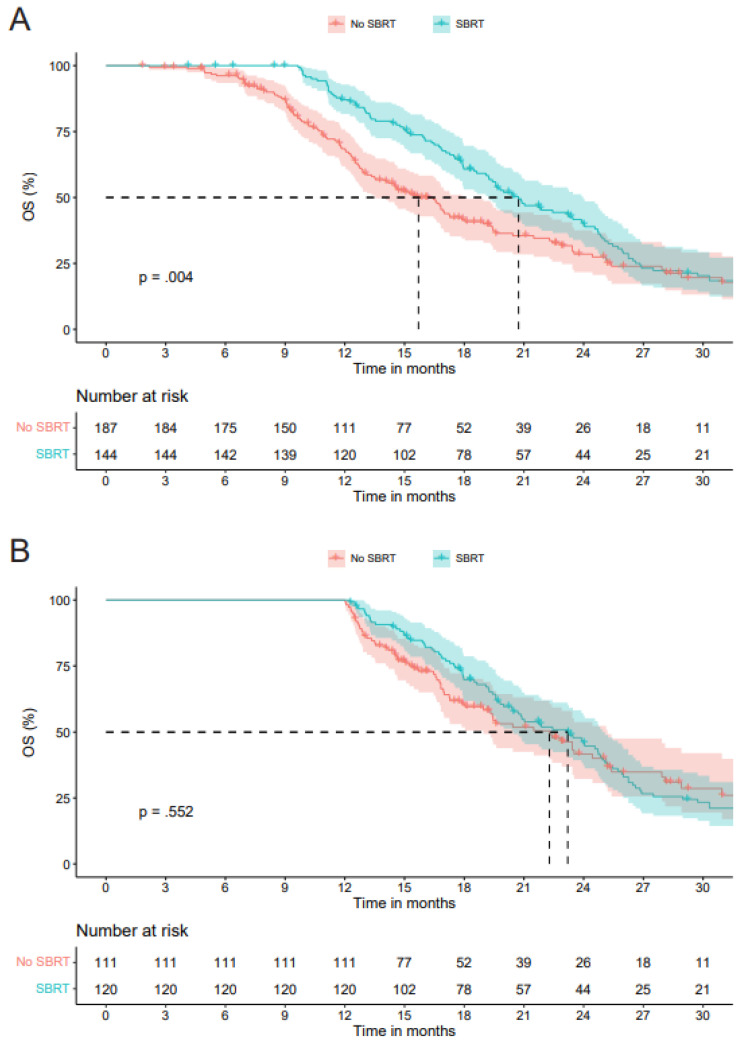
(**A**) Kaplan–Meier curves showing overall survival probability in the overall cohort, comparing the SBRT group with the No SBRT group. (**B**) Kaplan–Meier curves showing overall survival probability in the landmark analysis, comparing the SBRT group with the No SBRT group.

In the overall cohort (n = 331), at a median follow-up of 31.0 months (95%CI 25.5–36.4), 228 (68.9%) patients had died. The mOS of all patients was 18.0 months (95%CI 16.5–19.5). The mOS was 20.7 months (95%CI 18.5–23.0) in the SBRT group versus 15.7 months (95%CI 13.7–17.7) in the No SBRT group (*p* = 0.004) (Figure 2A).

#### 3.2.2. Overall Survival—Landmark Analysis

In the landmark analysis (n = 231), at a median follow-up time of 35.2 months (95%CI 29.4–41.0), 156 (67.5%) patients had died. The mOS of all patients was 22.4 months (95%CI 20.3–24.5). The mOS was 23.2 months (95%CI 20.5–25.9) in the SBRT group versus 22.3 months (95%CI 18.9–25.6) in the No SBRT group (*p* = 0.552) (Figure 2B).

#### 3.2.3. Overall Survival in Non-Resected Cohort—Landmark Analysis

**Figure 3:**
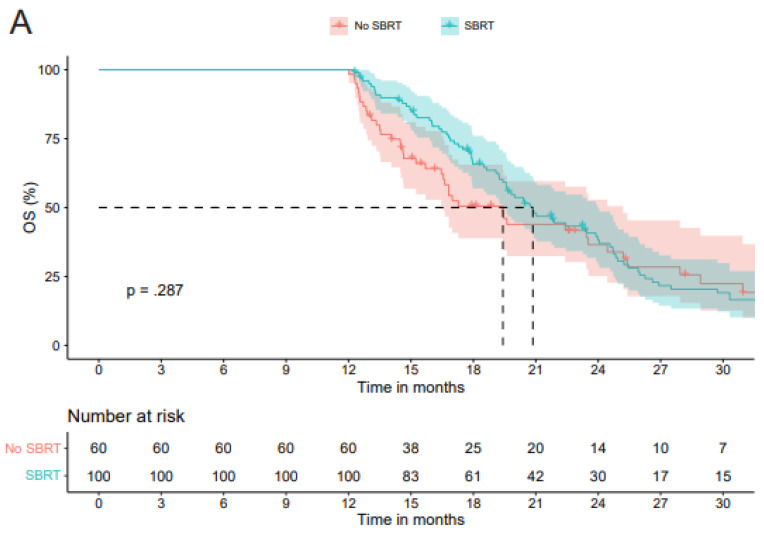

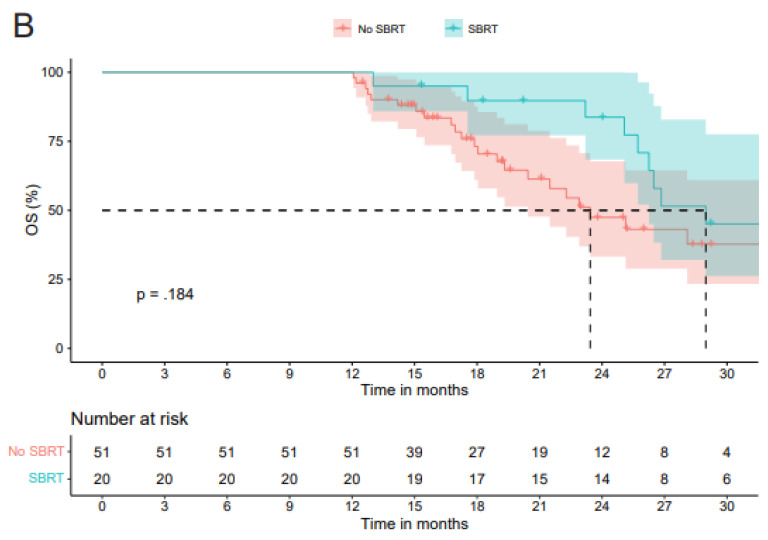
(**A**) Kaplan–Meier curves showing overall survival probability in the non-resected cohort in the landmark analysis, comparing the SBRT group with the No SBRT group. (**B**) Kaplan–Meier curves showing overall survival probability in the resected cohort in the landmark analysis, comparing the SBRT group with the No SBRT group.

In the non-resected cohort in the landmark analysis (n = 160), at a median follow-up of 36.0 months (95%CI 31.4–40.5), 124 (77.5%) patients had died. The mOS of all patients was 20.0 months (95%CI 18.1–21.8). The mOS was 20.9 months (95%CI 18.9–22.9) in the SBRT group versus 19.4 months (95%CI 16.6–22.2) in the No SBRT group (*p* = 0.287) (Figure 3A).

#### 3.2.4. Overall Survival in Resected Cohort—Landmark Analysis

In the resected cohort in the landmark analysis (n = 71), at a median follow-up of 28.4 months (95%CI 23.8–33.0), 32 (45.1%) patients had died. The mOS of all patients was 26.3 months (95%CI 24.3–28.2). The mOS was 29.0 months (95%CI 20.0–37.9) in the SBRT group compared to 23.4 months (95%CI 19.3–27.6) in the No SBRT group (*p* = 0.184) (Figure 3B). The mOS from resection was 18.6 months (95%CI 13.1–24.2) in the SBRT group compared to 19.7 months (95%CI 15.7–23.8) in the No SBRT group (*p* = 0.717) (Supplementary Figure S1, online material).

#### 3.2.5. Variables Associated with Overall Survival—Landmark Analysis

Cox regression analysis for OS in the landmark analysis cohort revealed that in univariable analysis, a baseline CA 19-9 ≥ 500 U/mL was associated with a decreased OS (HR 1.539; [95%CI 1.073–2.207]; *p* = 0.019). Treatment with (m)FOLFIRINOX-only without SBRT was not associated with OS (HR 1.103; [95%CI 0.799–1.523]; *p* = 0.553). In the multivariable analysis, baseline CA 19-9 and the number of cycles of neoadjuvant/induction (m)FOLFIRINOX were included. Baseline CA 19-9 ≥ 500 U/mL was independently associated with poor OS (HR 1.490; [95%CI 1.036–2.142]; *p* = 0.031). Moreover, ≥ 5 cycles of neoadjuvant/induction (m)FOLFIRINOX was not independently associated with OS (HR 1.418; [95%CI 0.855–2.352]; *p* = 0.176) (Table 2).

### 3.3. Secondary Outcomes

#### Histopathological Characteristics in Resected Cohort—Landmark Analysis

Factors associated with the SBRT group compared to the No SBRT group were: ypT0-2 in 95% versus 76.5% (*p* = 0.026), ypN0 in 75% versus 37.3% (*p* = 0.004), and absence of perineural invasion in 50% versus 68.6% (*p* = 0.015) (Table 3).

## 4. Discussion

This study explored the potential additional value of SBRT in patients with PDAC with vascular contact after at least four cycles of (m)FOLFIRINOX. In the overall cohort, 144 patients were included in the SBRT group, and 187 patients were included in the No SBRT group. The mOS was 20.7 months in the SBRT group versus 15.7 months in the No SBRT group (*p* = 0.004). In the landmark analysis, 120 patients were included in the SBRT group, and 111 patients were included in the No SBRT group. There was no difference in mOS between both groups: 23.2 months in the SBRT group versus 22.3 months in the No SBRT group (*p* = 0.552). Compared to treatment with (m)FOLFIRINOX alone, combination treatment of (m)FOLFIRINOX and SBRT was associated with more ypT0-2 and ypN0, and less perineural invasion. However, these favorable histopathological findings did not translate into prolonged survival after SBRT in the resected cohort. In the resected cohort, the mOS calculated from surgery was 18.6 months in the SBRT group compared to 19.7 months in the No SBRT group (*p* = 0.717).

A recent randomized trial, including 126 patients with BRPC, sought to determine the potential added value of SBRT after (m)FOLFIRINOX treatment [14]. The study comprised two arms: eight cycles of (m)FOLFRIINOX (arm 1) versus seven cycles of (m)FOLFIRINOX followed by 33–40 Gray of SBRT (arm 2). In arm 1, 70 patients were included, and in arm 2, 56 patients were included. Due to too few R0 resections in arm 2, patient accrual was terminated early in arm 2 as required per protocol. The study found an mOS of 29.8 months (95%CI 21.1–36.6) in arm 1 and 17.1 months (95%CI 12.8–24.4) in arm 2. Because patient accrual in arm 2 was terminated early, no statistical inference for efficacy was performed. However, the OS difference was notable, and the authors concluded that eight cycles of (m)FOLFIRINOX without consecutive SBRT should be considered the standard regimen for patients with BRPC.

Even though it was a randomized trial, the proportions of patients undergoing a resection (49% versus 35%), starting adjuvant FOLFOX (34% versus 24%), and completing the total trial treatment (31% versus 18%) were all higher in the (m)FOLFIRINOX-only group (arm 1).

Several single-armed cohort studies have explored the value of SBRT in localized PDAC [6,7,8,9,10,11,12,13]. The authors of some of these studies concluded that SBRT effectively prevented local disease progression [6,8,9]. These studies also reported high R0 resection rates (ranging from 75 to 100%) [6,9,11,12,13] and node-negative disease rates (ranging from 51 to 86%) [11,12,13] following SBRT treatment. In the PREOPANC trial, which demonstrated improved OS after neoadjuvant gemcitabine-based radiotherapy, R1 resection rate, pathologic lymph nodes, perineural invasion, and vascular invasion were all less frequent in the neoadjuvant chemoradiotherapy group [22]. These data align with that of the present study, in which we also found favorable histopathological characteristics in the SBRT group. Besides a possible anti-cancer effect, superior histopathological characteristics after radiation may be partly due to immortal time bias. In another study, around 13% of patients developed metastatic disease at restaging after completion of radiation therapy [23]. Consequently, those patients were not included in the resected cohort of the SBRT group. The landmark analysis, however, should have accounted for this bias.

The question remains, however, why the possible superior histopathological characteristics did not translate into a longer OS in the SBRT group. Possible explanations regarding this question are, first, that SBRT induces only a local effect without the ability to inhibit the progression of distant metastases. This is problematic since even in the earliest stages, PDAC is generally considered to be a systemic disease [24,25], and metastases are regarded as the most important cause of death in patients with PDAC [26,27,28]. It is likely that distant micrometastatic progression remains the dominant determinant of survival in this population. Second, several factors could have been in favor of the No SBRT group. For example, in the SBRT group, 16.7% of patients underwent a resection compared to 45.9% in the No SBRT group (*p* < 0.001). Also, the total number of cycles of (m)FOLFIRINOX was lower in the SBRT group (median 8 versus 9 cycles, *p* = 0.020). This difference is so small, however, that the impact on influencing OS might be negligible. On the other hand, in the SBRT group, 37.5% underwent a staging laparoscopy compared to 2.7% in the No SBRT group (*p* < 0.001). These laparoscopies were not performed because tumors were considered to be more advanced. Of the SBRT group, 21.2% participated in the LAPC-1 trial, and 22.6% participated in the LAPC-2 trial [11,12]. A staging laparoscopy was part of those trials and was done at the express request of the radiotherapist in order to optimize staging for eventual SBRT treatment. This suggests that the SBRT group was staged more accurately, since the yield of occult metastatic disease from staging laparoscopy has been reported to be as high as 20% [29,30].

This study had several limitations. First, confounding by indication may be present in this retrospective study, as specific patient and disease characteristics could have led to the allocation of a patient to SBRT or resection. In fact, the higher proportion of resections in the No SBRT group might reflect this bias. It is likely that patients with anatomically more complex tumors were allocated to SBRT treatment since radical resection of such a tumor was deemed difficult or impossible to achieve. Yet, the extent of blood vessel contact was equal between both groups, which pleads against this hypothesis. Also, possibly more inoperable patients were allocated to SBRT treatment since ECOG 0 was less prevalent in the SBRT group. However, all consecutive patients who were referred to the Erasmus MC Cancer Center and who were possible candidates for SBRT were offered SBRT treatment. Second, the SBRT group might have been subject to immortal time bias. Early development of metastases after chemotherapy would have precluded subsequent locoregional treatment. However, this limitation should have been addressed by the landmark analysis. Third, the fact that SBRT was given at one institution may have introduced institutional and referral bias.

Currently, emerging techniques are being used to deliver a biological equivalent dose of 100 Gray to the tumor. Future research is needed to investigate whether stereotactic radiotherapy with these ablative dosages can be delivered safely to pancreatic tumors and is able to improve outcomes. The LAPSTAR trial, an upcoming randomized trial by the Dutch Pancreatic Cancer Group, will seek to obtain evidence of the added value of MR-guided radiotherapy on health-related quality of life in patients with LAPC after initial systemic therapy.

## 5. Conclusions

In a landmark analysis, including only patients who survived for at least 12 months after diagnosis, we found no difference in median OS after treatment with (m)FOLFIRINOX-only and (m)FOLFIRINOX with consecutive SBRT.

## Figures and Tables

**Table 1:** Patient, disease and treatment characteristics—Landmark analysis cohort.

	Overall *n* = 231 (100%)	SBRT *n* = 120 (51.9%)	No SBRT *n* = 111 (48.1%)	*p*-Value
Patient characteristics				
Male sex, *n* (%)	115 (49.8)	61 (50.8)	54 (48.6)	0.740 ^a^
Age (years), median (IQR)	63 (57–71)	63 (57–71)	64 (57–70)	0.986 ^b^
ECOG-PS, *n* (%)				0.005^a^
ECOG 0	102 (44.2)	44 (36.7)	58 (52.3)	
ECOG 1	95 (41.1)	59 (49.2)	36 (32.4)	
ECOG 2	12 (5.2)	3 (2.5)	9 (8.1)	
Missing	22 (9.5)	14 (11.6)	8 (7.2)	
BMI (kg/m^2^), median (IQR)	24 (22–27)	24 (22–26)	24 (22–26)	0.543 ^b^
Missing, *n* (%)	49 (21.2)	22 (18.3)	27 (24.3)	
Disease characteristics				
Tumor location, *n* (%)				0.983 ^a^
Head	150 (64.9)	78 (65)	72 (64.9)	
Body/tail	81 (35.1)	42 (35)	39 (35.1)	
Tumor size (mm), median (IQR)	38 (30–47)	39 (31–47)	37 (30–48)	0.759 ^b^
Missing, *n* (%)	10 (4.3)	6 (5)	4 (3.6)	
Arterial blood vessel contact, *n* (%)				0.169 ^a^
≤180°	92 (39.8)	41 (34.2)	51 (46)	
>180°	118 (51.1)	68 (56.7)	50 (45)	
None	20 (8.7)	10 (8.3)	10 (9)	
Missing	1 (0.4)	1 (0.8)	0 (0)	
Venous blood vessel contact, *n* (%)				0.629 ^a^
≤270°	109 (47.2)	57 (47.5)	52 (46.8)	
>270°	74 (32.0)	34 (28.3)	40 (36)	
None	39 (16.9)	21 (17.5)	18 (16.2)	
Missing	9 (3.9)	8 (6.7)	1 (0.9)	
NCCN stage, *n* (%)				0.738 ^a^
LAPC	142 (61.5)	75 (62.5)	67 (60.4)	
BRPC	89 (38.5)	45 (37.5)	44 (39.6)	
CA 19-9 (U/mL, diagnosis), median (IQR)	290 (92–1134)	356 (94–1336)	268 (69–681)	0.159 ^b^
Missing, *n* (%)	23 (10)	5 (4.2)	18 (16.2)	
Treatment characteristics				
Staging laparoscopy, *n* (%)	48 (20.8)	45 (37.5)	3 (2.7)	<0.001 ^a^
Number of cycles of neoadjuvant/induction FOLFIRINOX, median (IQR)	8 (5–8)	8 (8–8)	5 (4–9)	<0.001 ^b^
Resection, *n* (%)	71 (30.7)	20 (16.7)	51 (45.9)	<0.001 ^a^
Adjuvant chemotherapy, *n* (%)	38 (53.5)	0 (0)	38 (74.5)	<0.001 ^a^
FOLFIRINOX, n (%)	35 (92.1)	0 (0)	35 (92.1)	-
Gemcitabine, n (%)	1 (2.6)	0 (0)	1 (2.6)	-
Unknown, n (%)	2 (5.3)	0 (0)	2 (5.3)	-
Number of cycles of adjuvant FOLFIRINOX, median (IQR)	6 (4–8)	0 (0)	6 (4–8)	-
Total number of cycles FOLFIRINOX, median (IQR)	8 (8–11)	8 (8–8)	9 (6–12)	0.020 ^b^
Interval stop FOLFIRINOX—start SBRT (weeks), median (IQR)	-	9 (7–11)	-	-
Interval diagnosis—start SBRT (months), median (IQR)	-	7 (6–8)	-	-

^a^, Pearson’s chi-squared test; ^b^, Mann–Whitney U test; SBRT, stereotactic body radiotherapy; IQR, interquartile range; ECOG-PS, Eastern Cooperative Oncology Group Performance Status; BMI, body mass index; NCCN, National Comprehensive Cancer Network; LAPC, locally advanced pancreatic cancer; BRPC, borderline resectable pancreatic cancer; CA, carbohydrate antigen.

**Table 2:** Cox regression analysis in landmark analysis cohort—Overall survival.

	Univariable Analysis			Multivariable Analysis		
Variable	HR	95%CI	*p*	HR	95%CI	*p*
Age (years)	0.998	0.981–1.015	0.774	**-**	**-**	**-**
Sex						
Female (n = 116)	1	[Referent]		**-**	**-**	**-**
Male (n = 115)	1.058	0.773–1.448	0.726	**-**	**-**	**-**
ECOG-PS						
0 (n = 102)	1	[Referent]		**-**	**-**	**-**
≥1 (n = 107)	1.231	0.883–1.717	0.220	**-**	**-**	**-**
Tumor location						
Body/tail (n = 81)	1	[Referent]		**-**	**-**	**-**
Head (n = 150)	1.002	0.724–1.388	0.988	**-**	**-**	**-**
Tumor size (mm)	1.005	0.993–1.017	0.435	**-**	**-**	**-**
Staging laparoscopy (diagnosis)						
Yes (n = 48)	1	[Referent]		**-**	**-**	**-**
No (n = 183)	1.138	0.801–1.617	0.470	**-**	**-**	**-**
CA 19-9 (U/mL, diagnosis)						
<500 (n = 97)	1	[Referent]		1	[Referent]	
≥500 (n = 81)	1.539	1.073–2.207	0.019	1.490	1.036–2.142	0.031
Number of cycles of neoadjuvant/induction CTx						
4 (n = 54)	1	[Referent]		1	[Referent]	
≥5 (n = 177)	1.355	0.876–2.096	0.172	1.418	0.855–2.352	0.176
SBRT						
Yes (n = 120)	1	[Referent]		**-**	**-**	**-**
No (n = 111)	1.103	0.799–1.523	0.553	**-**	**-**	**-**

HR, hazard ratio; CI, confidence interval; ECOG-PS, Eastern Cooperative Oncology Group Performance Score; CA, carbohydrate antigen; CTx, chemotherapy.

**Table 3:** Histopathological characteristics—Landmark analysis cohort.

	Overall n = 71 (100%)	SBRT n = 20 (28.2%)	No SBRT n = 51 (71.8%)	*p*-Value
Tumor size (mm), median (IQR)	25 (14–35)	7 (0–25)	28 (21–39)	<0.001 ^a^
ypT stage, n (%)				0.026 ^b^
T0-2	58 (81.7)	19 (95)	39 (76.5)	
T3-4	11 (15.5)	0 (0)	11 (21.6)	
Missing	2 (2.8)	1 (5)	1 (2)	
ypN stage, n (%)				0.004 ^b^
N0	34 (47.9)	15 (75)	19 (37.3)	
N1-2	37 (52.1)	5 (25)	32 (62.7)	
R-status, n (%)				0.093 ^b^
R0	46 (64.8)	16 (80)	30 (58.8)	
R1	25 (35.2)	4 (20)	21 (41.2)	
Perineural invasion, n (%)				0.015 ^b^
Present	41 (57.7)	6 (30)	14 (27.5)	
Absent	24 (33.8)	10 (50)	35 (68.6)	
Missing	6 (8.5)	4 (20)	2 (3.9)	
Lymphovascular invasion, n (%)				0.093 ^b^
Present	22 (31.0)	3 (15)	19 (37.3)	
Absent	39 (54.9)	13 (65)	26 (51)	
Missing	10 (14.1)	4 (20)	6 (11.7)	

^a^, Mann–Whitney U test; ^b^ Pearson’s chi-squared test; SBRT, stereotactic body radiotherapy; TNM staging according to the *AJCC Cancer Staging Manual*, 8th edition; R0, tumor clearance > 1 mm; R1, tumor clearance ≤ 1 mm.

## Data Availability

The raw data supporting the conclusions of this article will be made available by the corresponding author on request.

## Supplementary Materials

The following supporting information can be downloaded at: https://www.mdpi.com/article/10.3390/cancers18040700/s1, Supplementary Figure S1, online material: Kaplan–Meier curves showing overall survival probability in the resected cohort in the landmark analysis, comparing the SBRT group with the No SBRT group. Overall survival was calculated from the date of resection. Supplementary Table S1, online material: Patient, disease and treatment characteristics—Overall cohort. Supplementary Table S2, online material: Patient, disease and treatment characteristics of the resected cohort—Landmark analysis cohort.
