# Supplementary material for: The Value of Stereotactic Radiotherapy After FOLFIRINOX in Patients with Pancreatic Cancer with Vascular Contact—A Nationwide, Retrospective Cohort Study
Source: Cancers (Basel). 2026 Feb 20;18(4):700. doi: 10.3390/cancers18040700 (PMC12939309; doi:10.3390/cancers18040700)
Supplement: Supplementary file 1 [file cancers-18-00700-s001.zip › Table S1 - Demographics overall cohort.pdf]

Supplementary Table S1. Patient, disease and treatment characteristics - Overall cohort

	Overall n=331 (100%)	SBRT n=144 (43.5%)	No SBRT n=187 (56.5%)	p-value
**Patient characteristics**				
Male sex, n (%)	172 (52)	78 (54.2)	94 (50.3)	.481[a]
Age (years), median (IQR)	64 (56 – 70)	63 (56 – 71)	64 (56 – 70)	.697[b]
ECOG-PS, n (%)				**<.001[a]**
ECOG 0	140 (42.3)	48 (33.3)	92 (49.2)	
ECOG 1	136 (41.1)	74 (51.4)	62 (33.2)	
ECOG 2	14 (4.2)	3 (2.1)	11 (5.9)	
Missing	41 (12.4)	19 (13.2)	22 (11.7)	
BMI (kg/m^2), median (IQR)	24 (22 – 26)	25 (22 – 28)	24 (22 – 26)	.191[b]
Missing, n (%)	81 (24.5)	29 (20.1)	52 (27.8)	
**Disease characteristics**				
Tumor location, n (%)				.611[a]
Head	216 (65.3)	97 (67.4)	119 (63.6)	
Body/tail	112 (33.8)	47 (32.6)	65 (34.8)	
Missing	3 (0.9)	0 (0)	3 (1.6)	
Tumor size (mm), median (IQR)	39 (30 – 48)	39 (31 – 47)	38 (30 – 50)	.861[b]
Missing, n (%)	17 (5.1)	7 (4.9)	10 (5.3)	
Arterial blood vessel contact, n (%)				.428[a]
≤ 180°	132 (39.9)	52 (36.1)	80 (42.8)	
> 180°	169 (51.1)	79 (54.9)	90 (48.1)	
None	26 (7.9)	12 (8.3)	14 (7.5)	
Missing	4 (1.2)	1 (0.7)	3 (1.6)	
Venous blood vessel contact, n (%)				.487[a]
≤ 270°	152 (45.9)	70 (48.6)	82 (43.9)	
> 270°	107 (32.3)	42 (29.2)	65 (34.8)	
None	58 (17.5)	23 (16.0)	35 (18.7)	
Missing	14 (4.2)	9 (6.2)	5 (2.7)	
NCCN-stage, n (%)				.752[a]
LAPC	206 (62.2)	91 (63.2)	115 (61.5)	
BRPC	125 (37.8)	53 (36.8)	72 (38.5)	
CA 19-9 (U/ml, diagnosis), median (IQR)	274 (64 – 1200)	354 (91 – 1390)	242 (48 – 695)	**.040[b]**
Missing, n (%)	38 (11.5)	8 (5.6)	30 (16)	
**Treatment characteristics**				
Staging laparoscopy, n (%)	59 (17.8)	53 (36.8)	6 (3.2)	**<.001[a]**
Number of cycles neoadjuvant/induction FOLFIRINOX, median (IQR)	8 (4 – 8)	8 (8 – 8)	5 (4 – 8)	**<.001[b]**
Resection, n (%)	89 (26.9)	21 (14.6)	68 (36.4)	**<.001[a]**
Adjuvant chemotherapy, n (%)	47 (52.8)	0 (0)	47 (69.1)	**<.001[a]**
FOLFIRINOX, n (%)	44 (93.6)	0 (0)	44 (93.6)	-
Gemcitabine, n (%)	1 (2.1)	0 (0)	1 (2.1)	-
Unknown, n (%)	2 (4.3)	0 (0)	2 (4.3)	-
Number of cycles of adjuvant FOLFIRINOX, median (IQR)	6 (4 – 8)	0 (0)	6 (4 – 8)	-
Total number of cycles FOLFIRINOX, median (IQR)	8 (5 – 9)	8 (8 – 8)	8 (4 – 12)	.442[b]
Interval stop FOLFIRINOX – start SBRT (weeks), median (IQR)	-	9 (7 – 12)	-	-
Interval diagnosis – start SBRT (months), median (IQR)	-	7 (6 – 8)	-	-

a, Pearson Chi-Squared test; b, Mann-Whitney U test; SBRT, stereotactic body radiotherapy; IQR, interquartile range; ECOG-PS, Eastern Cooperative Oncology Group Performance Status; BMI, body mass index; NCCN, National Comprehensive Cancer Network; LAPC, locally advanced pancreatic cancer; BRPC, borderline resectable pancreatic cancer; CA, carbohydrate antigen
