# Supplementary material for: The Value of Stereotactic Radiotherapy After FOLFIRINOX in Patients with Pancreatic Cancer with Vascular Contact—A Nationwide, Retrospective Cohort Study
Source: Cancers (Basel). 2026 Feb 20;18(4):700. doi: 10.3390/cancers18040700 (PMC12939309; doi:10.3390/cancers18040700)
Supplement: Supplementary file 1 [file cancers-18-00700-s001.zip › Table S2 - Demographics of resected cohort.pdf]

Supplementary Table S2. Patient, disease and treatment characteristics of the resected cohort – Landmark analysis cohort

	**Overall n=71 (100%)**	**SBRT n=20 (28.2%)**	**No SBRT n=51 (71.8%)**	**p-value**
**Patient characteristics**				
Male sex, n (%)	31 (43.7)	9 (40.7)	22 (43.1)	.887[a]
Age (years), median (IQR)	63 (54 – 70)	56 (50 – 63)	66 (58 – 70)	**.003**[b]
ECOG-PS, n (%)				**.041**[a]
ECOG 0	38 (53.5)	6 (30)	32 (62.7)	
ECOG 1	26 (36.6)	11 (55)	15 (29.4)	
ECOG 2	2 (2.8)	0 (0)	2 (3.9)	
Missing	5 (7)	3 (15)	2 (3.9)	
BMI (kg/m^2), median (IQR)	24 (22 – 26)	25 (20 – 27)	24 (22 – 25)	.498[b]
Missing, n (%)	21 (29.6)	5 (25)	16 (31.4)	
**Disease characteristics**				
Tumor location, n (%)				.331[a]
Head	59 (83.1)	18 (90)	41 (80.4)	
Body/tail	12 (16.9)	2 (10)	10 (19.6)	
Tumor size at diagnosis (mm), median (IQR)	36 (30 – 45)	40 (33 – 47)	36 (30 – 42)	.258[b]
Missing, n (%)	4 (5.6)	0 (0)	4 (7.8)	
Arterial blood vessel contact, n (%)				.476[a]
≤ 180°	40 (56.3)	9 (45)	31 (60.8)	
> 180°	22 (31.0)	8 (40)	14 (27.5)	
None	9 (12.7)	3 (15)	6 (11.8)	
Venous blood vessel contact, n (%)				.417[a]
≤ 270°	40 (56.3)	9 (45)	31 (60.8)	
> 270°	15 (21.1)	6 (30)	9 (17.6)	
None	16 (22.5)	5 (25)	11 (21.6)	
NCCN-stage, n (%)				.601[a]
LAPC	32 (45.1)	10 (50)	22 (43.1)	
BRPC	39 (54.9)	10 (50)	29 (56.9)	
CA 19-9 (U/ml, diagnosis), median (IQR)	238 (67 – 984)	325 (105 – 1200)	231 (46 – 474)	.310[b]
Missing, n (%)	9 (12.7)	1 (5)	8 (15.7)	
**Treatment characteristics**				
Staging laparoscopy, n (%)	11 (15.5)	10 (20)	1 (2.0)	**<.001**[a]
Number of cycles neoadjuvant/induction FOLFIRINOX, median (IQR)	5 (4 - 8)	8 (8 – 8)	4 (4 – 5)	**<.001**[b]
Adjuvant chemotherapy, n (%)	38 (53.5)	0 (0)	38 (74.5)	**<.001**[a]
FOLFIRINOX, n (%)	35 (92.1)	0 (0)	35 (92.1)	-
Gemcitabine, n (%)	2 (5.3)	0 (0)	1 (2.6)	-
Unknown, n (%)	1 (2.6)	0 (0)	2 (5.3)	-
Number of cycles of adjuvant FOLFIRINOX, median (IQR)	6 (4 – 8)	0	6 (4 – 8)	-
Total number of cycles FOLFIRINOX, median (IQR)	8 (8 – 12)	8 (8 – 8)	11 (7 – 12)	.076[b]

a, Pearson Chi-Squared test; b, Mann-Whitney U test; SBRT, stereotactic body radiotherapy; IQR, interquartile range; ECOG-PS, Eastern Cooperative Oncology Group Performance Status; BMI, body mass index; NCCN, National Comprehensive Cancer Network; LAPC, locally advanced pancreatic cancer; BRPC, borderline resectable pancreatic cancer; CA, carbohydrate antigen
