# Supplementary figures and images for: The Value of Stereotactic Radiotherapy After FOLFIRINOX in Patients with Pancreatic Cancer with Vascular Contact—A Nationwide, Retrospective Cohort Study
Source: Cancers (Basel). 2026 Feb 20;18(4):700. doi: 10.3390/cancers18040700 (PMC12939309; doi:10.3390/cancers18040700)

Figure S1

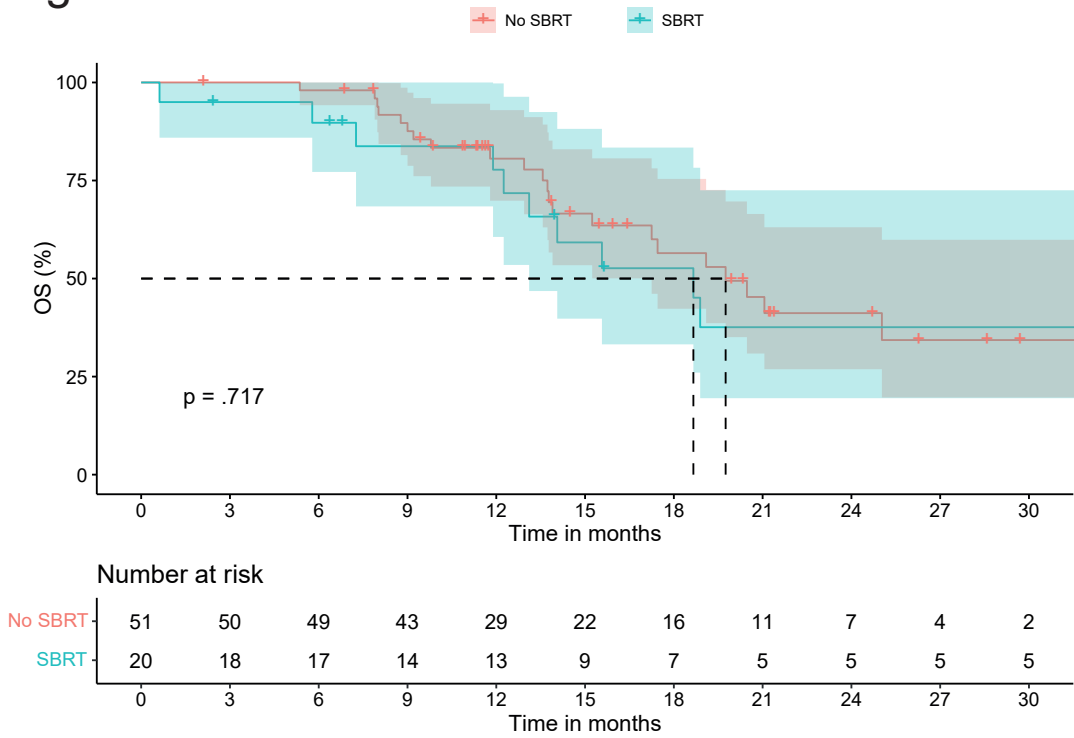

Supplement: Supplementary file 1 [file cancers-18-00700-s001.zip › Figure S1.pdf]
